# IL1B gene variants, but not TNF, CXCL8, IL6 and IL10, modify the course of cystic fibrosis in Polish patients.

**DOI:** 10.12688/f1000research.110472.3

**Published:** 2022-11-22

**Authors:** Oliwia Zakerska-Banaszak, Joanna Gozdzik-Spychalska, Marcin Gabryel, Joanna Zuraszek, Marzena Skrzypczak-Zielinska, Ryszard Slomski, Agnieszka Dobrowolska, Tomasz Piorunek, Halina Batura-Gabryel

**Affiliations:** 1 Institute of Human Genetics Polish Academy of Sciences, Poznan, Poland; 2 Department of Pulmonology, Allergology and Lung Oncology, Poznan University of Medical Sciences, Poznań, Poland; 3 Department of Gastroenterology, Dietetics and Internal Medicine, Poznan University of Medical Sciences, Poznan, Poland

**Keywords:** cystic fibrosis, modifier genes, inflammatory mediators, IL1B

## Abstract

***Background:*** The main aim of this study was to evaluate whether selected polymorphic variants in genes from the inflammatory pathway can be predictors of pulmonary or digestive manifestation of cystic fibrosis, as well as of severity of lung disease.

***Materials and methods:*** Using pyrosequencing and sequencing we have genotyped 12 variants in *TNF* (rs361525, rs1800629), *CXCL8* (rs4073, rs2227306, rs2227307, rs188378669), *IL1B* (rs16944, rs1143634, rs1142639, rs1143627), *IL6* (rs1800795) and *IL10* (rs1800896) genes in a cohort of 55 Polish patients with diagnosed cystic fibrosis and controls. In our study group, a pulmonary manifestation of disease revealed 44 of subjects (80%), and digestive symptoms dominated in 11 (20%) of analyzed individuals. Severe lung dysfunction has occurred in 20 (36.4%) of patients.

***Results:*** We proved, that two promoter variants of *IL1B,* rs1143627 (c.-118G > A) and rs16944 (c.-598T > C) are presented significantly more often in patients with severe character of lung disease compared to mild (82.5% *vs.* 62.8%, p-value 0.030, and 87.5% *vs.* 64.3%, p-value 0.008, respectively) in cystic fibrosis course. Haplotype AC formed by both changes had also a higher frequency (80%) in patients with severe course compared to the mild character (61.4%) of disease. However, the frequency of promoter variant *TNF* c.-308C > T (rs1800629) was presented at a significantly lower level in the patient’s group compared to healthy controls (2.7% *vs.* 15%, p-value 0.001). Furthermore, the presence of methicillin-resistant *Staphylococcus aureus* significantly correlated with the lower FEV1% in patients (p-value 0.01).

***Conclusions:*** Genetic variants, rs1143627 and rs16944, of *IL1B* are promising candidates as predictors of the severe character of lung disease in Polish patients with cystic fibrosis.

## Introduction

Recent scientific outcomes confirm that the clinical phenotype of cystic fibrosis (CF) (OMIM: 219700) is determined not only by classes of mutations in the *CFTR* gene (cystic fibrosis transmembrane regulator) but in association with environmental factors and genetic variations in modifier genes. ^1^^–^^3^ The hypothesis about the role of modifier genes in CF was born based on the observations, that patients with the same *CFTR* genotype presented diverse manifestations and course of the disease. ^4^ Today, over 2000 different *CFTR* mutations have been reported and F508del is by far the most common. ^5^ Although mutations in the *CFTR* gene are well known and classified, the contribution of modulatory genes in CF is currently still investigated. Among analyzed candidate genes are those involved in the inflammatory process, as well as in immunity and antioxidant molecules. ^6^ However, the results of the majority of global research on modifier genes’ role in CF are inconclusive.

CF is a multi-organ disease, whereas chronic pulmonary inflammation and respiratory failure consist of the main cause of death in those patients. There is evidence, that the inflammatory process in the lung is associated with an imbalance between pro- and anti-inflammatory mediators. ^7^ Among important pro-inflammatory cytokines produced during the response are tumor necrosis factor-alpha (TNF-α), interleukins (IL) 8, 6, 1, and 1B, while among cytokines inducing the opposite effect are transforming growth factor-beta 1 (TGFB1) and IL10. ^8^^–^^10^

Proteins, IL8 and TNF-α, play a crucial role in the pathophysiology of CF lung disease due to their participation in the recruitment and activation of neutrophils on the respiratory epithelial surface, which is a primary component of the innate immune response. ^11^ Thus, genes *CXCL8* and *TNF* coding for those cytokines, which expression is regulated by sequence variants, are highlighted as potential modifier genes in the severity of lung disease in CF. Although numerous polymorphic variants have been described in the *CXCL8* gene, the association only between polymorphisms rs4073 (c.-251T>A), rs2227306 (c.781C>T), rs2227307 (c.396T>G), pulmonary function, and clinical severity markers in CF patients was confirmed in several studies. ^8^^,^^12^ The most often analyzed changes in the *TNF* gene are located in the promoter region, such as c.-238G>A (rs361525) having a variable effect on gene expression and c.-308A>G (rs1800629) associated with increased gene transcription, worst pulmonary function, and early pulmonary symptoms in patients with CF. ^13^^,^^14^ in contrast to studies performed by Schmitt-Grohé *et al.* ^15^ and Khorrami *et al.* ^16^ There is also proven, that some of *TNF* and *CXCL8* polymorphisms are associated with *Pseudomonas aeruginosa* ( *PA*) chronic colonization in CF patients. ^14^^,^^17^ Furthermore, modulating effects on the CF also have shown *IL1B* and *IL10* genes, where the most common SNPs were associated with severe lung disease in pediatric American and Australian populations. ^18^^,^^19^ Whereas, in French and German pediatric CF patients those results were not shared. ^8^

Results of up to now performed studies, searching for genes that modify the course and phenotype of CF, mostly concern the association with pulmonary exacerbation. However, based on our long-term observations of CF patients we state, that around 20% of CF patients manifest a pronounced exacerbation of symptoms from the digestive system. We hypothesized, that this possibly may be predicted by polymorphic changes at immunologically relevant genes. Within this context, we have selected 12 polymorphisms located in five genes *CXCL8* (rs4073, rs2227306, rs2227307, rs188378669), *TNF* (rs361525, rs1800629), *IL1B* (rs16944, rs1143634, rs1142639, rs1143627), *IL6* (rs1800795), and *IL10* (rs1800896) for correlation analysis, as candidate genetic modulators of the pulmonary or digestive manifestation and severity of the disease among Polish CF patients.

## Materials and methods

### Patients and clinical data

The study was approved by the local Ethics Committee of the University of Medical Sciences in Poznan, Poland (resolution no. 675/15), and all experiments were performed following the relevant guidelines and regulations of this Committee. Written informed consent was obtained from each patient. 55 Polish patients (20 males and 35 females) between the ages of 20-52 with diagnosed CF were enrolled for this study. The patient group was collected in 12 months (from January to December 2016) in the Department of Pulmonology, Allergology and Lung Oncology of the Clinical Hospital of Poznan University of Medical Sciences in Poland. Diagnosis of CF in all patients was performed by sweat chloride test results (> 60 mmol/L) or/and identification of *CFTR* gene mutations. Detailed information about each patient including sex, age, BMI, age of diagnosis, presence of F508del mutation, pulmonary function parameters, function of internal organs, complications, and hospitalizations were recorded. Additionally, a control group of 50 healthy individuals was collected. A detailed characteristics of the study cohort with clinical and demographic data are presented in Table 1.

**Table 1.** Baseline characteristics of study participants.

Subjects characteristic	Patients n = 55	Controls n = 50
**Sex (M/F)**	20 (36%)/35 (64%)	19 (38%)/31 (62%)
**Age (years)**		
Mean (range)	28 (20-52)	31 (22-45)
**Ethnicity (Caucasian)**	55 (100%)	50 (100%)
**BMI**		
Mean (range)	20.2 (15-27.8)	23.3 (17.5-26)
Malnutrition	21 (38.2%)	2 (4%)
**Diagnosis (years)**		
Mean (range)	7.8 (1-36)	-
**Predominant manifestation of CF**		-
Pulmonary	44 (80%)	
Digestive	11 (20%)	
**CF genotype (F508del)**		-
Homozygote	13 (23.6%)	
Heterozygote	31 (56.4%)	
Nil	11 (20%)	
**Notably severe character of CF**	20 (36.4%)	-
**FEV1%**		-
Mean (range)	53.5 (10.6-106.8)	
**FVC%**		-
Mean (range)	67 (26.1-112.4)	
**TLC%**		-
Mean (range)	109.4 (78.7-151.6)	
**RV%**		-
Mean (range)	219.2 (88.8-391.6)	
**DLCO**		-
Mean (range)	59.7 (16.8-88)	
**Pulmonary complications** (emphysema, hemoptysis)	25 (45.5%)	-
**Lung transplantation/death**	14 (25.5%)	-
**Diabetes/impaired glucose tolerance**	29 (52.7%)	-
**Exocrine insufficiency**	38 (69%)	-
**Liver dysfunction**	19 (34.5%)	-
**Hospitalizations per year**		-
Mean (range)	1.9 (0-8)	

Pulmonary function tests, using Jaeger MasterScreen system (Erich Jaeger GmbH; Würzburg, Germany) were performed to assess lung function. All spirometric examinations were carried out with the subject seated, using a nose clip and a disposable mouthpiece. Using spirometric measurements, values of expiratory forced vital capacity (FVC) and forced expiratory volume in one second (FEV1%) were obtained and were expressed as the percentage of predicted values according to European Community for Steel and Coal. ^20^

At the same time the body plethysmography for assessing residual volume (RV), total lung capacity (TLC), and diffusing capacity of the lungs for carbon monoxide (DLCO) were performed.

Patients were divided in the context of lung function impairment, based on the FEV1% values, while they were clinically stable, 1 - within the norm (FEV1% ≥ 70) and mild pulmonary obstruction (FEV1% 40-70) (35 subjects in total), 2 - severe pulmonary obstruction (FEV1% ≤ 40) (20 subjects in total). ^21^ The “severe” group of patients did not differ significantly in age from patients in the “mild” group (mean age was 27.11 and 30.75, respectively; p-value was 0.055).

In an attempt to analyze the correlation between the genotype and manifestation of CF, patients were divided into two subgroups depending on the dominant symptoms - the group with the manifestation primarily from the respiratory system (44 individuals) and the group of patients with prevalent gastrointestinal symptoms (11 individuals). The division was made by the specialists from the pulmonology field conducting the patients, based on the clinical data and interview. To the digestive predominant phenotype were enrolled patients with the coexistence of at least two of listed conditions: 1) diabetes or glucose metabolism impairment, 2) pancreatic insufficiency, 3) liver disease or cirrhosis, 4) nagging pain or dysfunction of the digestive system. All patients with GI predominant phenotype represented “mild” lung impairment. Specialists determining the patients phenotype were not involved in the analysis of genotype assessment. Results of genotyping did not influence the assessment of phenotypic description.

### Genotyping

Genomic DNA of each patient was extracted from the peripheral blood samples (5 mL) using the standard method with guanidine isothiocyanate (GTC). Detection of the single nucleotide polymorphisms (SNPs) in five genes: *CXCL8* (rs4073, rs2227306, rs2227307, rs188378669), *TNF* (rs361525, rs1800629), *IL1B* (rs16944, rs1143634, rs1142639, rs1143627), *IL6* (rs1800795) and *IL10* (rs1800896) was performed using pyrosequencing or Sanger sequencing. Primers for the pyrosequencing analysis were designed using PyroMark Assay Design Software (Biotage, Uppsala, Sweden) and for Sanger sequencing using Primer3Plus software. Primer details are shown in Table 2. Amplification of targeted DNA regions was carried out on Applied Biosystems 2720 Thermal Cycler (Applied Biosystems, Foster City, CA) on the total volume of 30 uL containing 0.75 U of FIREPol® DNA Polymerase, 2.5 μL 10× buffer, 2.0 μL dNTP mix (2.5 mM each dNTP), 1.5 mM MgCl _2_ solution, 80 ng DNA and 0.2 μM of each primer. All reagents were obtained from Solis BioDyne (Tartu, Estonia). The amplification products were analyzed in 1.5% agarose gels electrophoresis. Pyrosequencing was performed by the PSQ™ 96MA system (Qiagen) using PyroMark™ Gold Q96 Reagents (Qiagen GmbH, Hilden, Germany), according to the manufacturer instructions. Direct sequencing was performed using BigDye Terminator v3.1 Cycle Sequencing Kit (Thermo Fisher Scientific) on the Applied Biosystems 3500 and Series Genetic Analyzers.

**Table 2.** Primer details.

Gene	SNP	Primer Name	Primer sequence	Product length
*TNF*	c.-238G > A (rs361525)	TNF_238_F *	5’-CTCCAGGGTCCTACACACAAAT-3’	188 bp
		TNF_238_R	5’-CATCTGGAGGAAGCGGTAGTG-3’	
		TNF_238_Seq	5’-CCCATCCTCCCTGCT-3’	-
	c.-308C > T (rs1800629)	TNF_308_F *	5’-GCCCCTCCCAGTTCTAGTTCT-3’	184 bp
		TNF_308_R	5’-ATTCCGAGGGGGGTCTTC-3’	
		TNF_308_Seq	5’-GGCTGAACCCCGTCC-3’	-
*CXCL8*	c.-251T > A (rs4073)	CXCL8_251_F	5’-ATCTTGTTCTAACACCTGCCACTC-3’	112 bp
		CXCL8_251_R *	5’-AAGCTCCACAATTTGGTGAATTA-3’	
		CXCL8_251_Seq	5’-TAGAAATAAAAAAGCATACA-3’	-
	c.781C > T (rs2227306)	CXCL8_781_F *	5’-GAAGGCAATTTCTATGCTGGAGAG-3’	225 bp
		CXCL8_781_R	5’-CCTGAATATTCTCCTAGCCCTTGA-3’	
		CXCL8_781_Seq	5’-CATAACTGACAACATTGAAC-3’	-
	c.396T > G (rs2227307)	CXCL8_396_F	5’-GCGTTTTCCTATGTCTAAATGTGA-3’	357 bp
		CXCL8_396_R *	5’-CAAATCTGAGGCTTGTCAATGA-3’	
		CXCL8_396_Seq	5’-CTGCTTTTATAATTTATACC-3’	-
	c.91G > T (rs188378669)	CXCL8_91_F	5’-ATCACTTTTTCCCCCAACAG-3’	246 bp
		CXCL8_91_R	5’-CCTAACACCTGGAACTTTCCTAAA-3’	
*IL1B*	c.-598T > C (rs16944)	IL1B_598_F *	5’-TGAGGGTGTGGGTCTCTACCTT-3’	112 bp
		IL1B_598_R	5’-AAGCTCCACAATTTGGTGAATTA-3’	
		IL1B_598_Seq	5’-TAGAAATAAAAAAGCATACA-3’	-
	c.315G > A (rs1143634)	IL1B _315_F	5’-CGTGCACATAAGCCTCGTTATC-3’	59 bp
		IL1B _315_R *	5’-GCTCCACATTTCAGAACCTATCTT-3’	
		IL1B _315_Seq	5’-CATAACTGACAACATTGAAC-3’	-
	c.597+76G > A (rs1142639)	IL1B _597_F *	5’-TTGAAGGTTGCACGCAGTTAA-3’	143 bp
		IL1B _597_R	5’-TCAGCCTCCTGCTACCACTTATT-3’	
		IL1B _597_Seq	5’-CAGACAACCACCTTCTC-3’	-
	c.-118G > A (rs1143627)	IL1B _118_F	5’-GTGCCTTGTGCCTCGAAGAG-3’	86 bp
		IL1B _118_R *	5’-TCAGCCTCCTACTTCTGCTTTTGA-3’	
		IL1B _118_Seq	5’-CCCTCGCTGTTTTTAT-3’	-
*IL6*	c.-237G > C (rs1800795)	IL6 _237_F *	5’-TGCACTTTTCCCCCTAGTTGT-3’	82 bp
		IL6 _237_R	5’-TGGGGCTGATTGGAAACCT-3’	
		IL6 _237_Seq	5’-TGTGACGTCCTTTAGCA-3’	-
*IL10*	c.-1117A > G (rs1800896)	IL10 _1117_F	5’-AACTGGCTCCCCTTACCTTCTA-3’	151 bp
		IL10 _1117_R *	5’-AGGCTGGATAGGAGGTCCCTTACT-3’	
		IL10_1117_Seq	5’-AAGGCTTCTTTGGGA-3’	-

* Primers labelled with biotin for pyrosequencing.

### Statistical analysis

Conformance of genotypes distribution of all analyzed polymorphisms with the Hardy-Weinberg equilibrium (HWE) was assessed using Fisher’s exact test. The pair-wise linkage disequilibrium (LD) of variants located in genes *TNF*, *CXCL8,* and *IL1B* was evaluated by Lewontin’s D′ using Haploview software version 4.2. The correlation analyses between genotypes and clinical data were performed using the chi-square test and Fisher’s exact test.

For all calculations, STATISTICA 12.0 software (Stat Soft, 2014) was used. The level of significance was set at p < 0.05.

## Results

### Allele frequencies and linkage disequilibrium analysis

A total of 55 CF Polish patients and 50 healthy controls were successfully genotyped for selected 12 polymorphisms located in genes *CXCL8*, *TNF*, *IL1B*, *IL6,* and *IL10.* Genotypes distribution for all SNPs met the requirements of HWE. No relevant differences in variant allele frequency between both groups were demonstrated. Only *TNF* c.-308C > T variant was observed significantly less often in the patient group (2.7%) compared to controls (15%), where the p-value was 0.001. All obtained frequencies of each genotype and allele are presented in Table 3.

**Table 3.** Genotypes and alleles distribution of analyzed polymorphisms among group of Polish CF patients and controls.

SNP	Genotype	Group of CF patients (n = 55)			Control group (n = 50)			1000 Genomes database	Allele frequency CF patients *vs* control group
		Number (%)	HWE ^**^ (p-value)	Variant allele freq.	Number (%)	HWE ^**^ (p-value)	Variant allele freq.	Variant allele freq. (EU population)	
*TNF* c.-238G > A (rs361525)	GG	48 (87.3)	0.614	6.4%	44 (88)	0.1	7%	6%	p = 0.853
	GA	7 (12.7)			5 (10)				
	AA	0 (0.0)			1 (2)				
*TNF* c.-308C > T (rs1800629)	CC	52 (94.5)	0.835	2.7%	36 (72)	0.889	15%	13%	**p = 0.001**
	CT	3 (5.5)			13 (26)				
	TT	0 (0.0)			1 (2)				
*CXCL8* c.-251T > A (rs4073)	TT	16 (29.1)	0.419	43.6%	17 (34)	0.916	42%	42%	p = 0.810
	TA	30 (54.5)			24 (48)				
	AA	9 (16.4)			9 (18)				
*CXCL8* c.781C > T (rs2227306)	CC	16 (29.1)	0.419	43.6%	17 (34)	0.916	42%	39%	p = 0.810
	CT	30 (54.5)			24 (48)				
	TT	9 (16.4)			9 (18)				
*CXCL8* c.396T > G (rs2227307)	TT	16 (29.1)	0.419	43.6%	17 (34)	0.916	42%	42%	p = 0.810
	TG	30 (54.5)			24 (48)				
	GG	9 (16.4)			9 (18)				
*CXCL8* c.91G > T (rs188378669)	GG	54 (98.2)	0.945	0.9%	50 (100)	-	0%	0%	p = 1.290
	GT	1 (1.8)			0 (0)				
	TT	0 (0.0)			0 (0)				
*IL1B* c.315G > A (rs1143634)	GG	34 (61.8)	0.311	20%	35 (70)	0.578	17%	25%	p = 0.576
	GA	20 (36.4)			13 (26)				
	AA	1 (1.8)			2 (4)				
*IL1B* c.-598T > C (rs16944)	TT	5 (9.1)	0.536	73%	5 (10)	0.736	70%	65%	p = 0.662
	TC	20 (36.4)			20 (40)				
	CC	30 (54.5)			25 (50)				
*IL1B* c.597+76G > A (rs1143639)	GG	34 (61.8)	0.311	20%	35 (70)	0.578	17%	24%	p = 0.576
	GA	20 (36.4)			13 (26)				
	AA	1 (1.8)			2 (4)				
*IL1B* c.-118G > A (rs1143627)	GG	5 (9.1)	0.974	70%	4 (8)	0.594	69%	65%	p = 0.875
	GA	23 (41.8)			23 (46)				
	AA	27 (49.1)			23 (46)				
*IL6* c.-237G > C (rs1800795)	GG	16 (29.1)	0.701	47%	20 (40)	0.406	39%	42%	p = 0.226
	GC	26 (47.3)			21 (42)				
	CC	13 (23.6)			9 (18)				
*IL10* c.1117A > G (rs1800896)	AA	16 (29)	0.185	43%	20 (40)	0.238	40%	45%	p = 0.688
	AG	31 (56.5)			20 (40)				
	GG	8 (14.5)			10 (20)				

^**^ HWE – Hardy-Weinberg equilibrium (occurs when p > 0.05).

**Figure 1.**
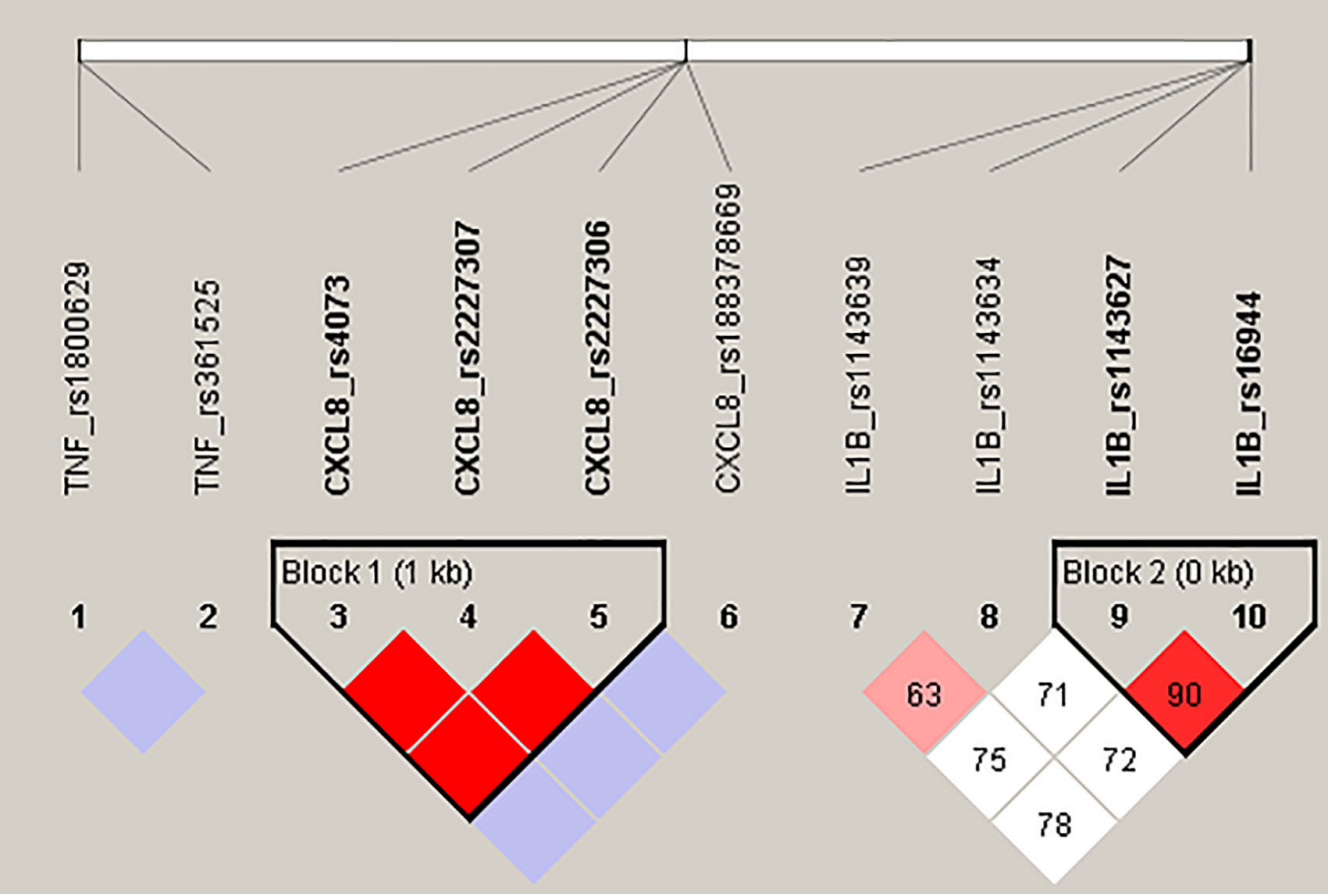
Detected haploblocks in CF patients.

Our haplotype analysis confirmed a strong LD (D *'* = 1, r ^2^ = 0.928) between variants c.-251T > A (rs4073), c.781C > T (rs2227306) and c.396T > G (rs2227307) in the *CXCL8* gene, forming four haplotypes: TCT, ATG, TCG and ACG observed in our CF patient group with frequency 54.6%, 41.8%, 1,8% and 1,8%, respectively. Furthermore, two polymorphic changes located in the promoter region of *IL1B* gene, c.-118G > A (rs1143627) and c.-598T > C (rs16944) were observed in high LD (D’ = 0.904, r ^2^ = 0.75), constructing a haploblock, where haplotypes AC, GT, GC, AT frequency was 68.1%, 26.3%, 3.7% and 1.9%, respectively. Both haploblocks are presented on the Figure 1.

### Association analysis of clinical data and genetic diversity

First, we have analyzed all 12 variants in designated, based on clinical data, groups of patients - with different manifestations of CF (with pulmonary or digestive dominant symptoms) and with variable courses of disease (mild or severe) to examine possible association.

Our study demonstrated that the presence of two polymorphisms, c.-598T > C (rs16944) and c.-118G > A (rs1143627), in *IL1B* gene significantly correlate with character of disease ( Table 4). Higher frequency of variant allele c.-598C was observed in patients with severe character of CF, compared to patients with mild course of disease (87.5% and 64.3%, respectively, χ ^2^ = 6.92; p = 0.008). Similarly, variant allele c.-118A occurred with higher frequency in subjects presented severe character of CF versus those with mild course of disease (82.5% vs. 62.8%, respectively, χ ^2^ = 4.68; p < 0.05).

**Table 4.** Distribution of analyzed polymorphisms in the studied group of CF patients with different manifestation and course of disease.

SNP	Genotype	CF manifestation					Course of disease				
		Pulmonary (n = 44)	Variant allele freq.	Digestive (n = 11)	Variant allele freq.	Pulmonary *vs.* Digestive	Mild (n = 35)	Variant allele freq.	Severe (n = 20)	Variant allele freq.	Mild *vs.* Severe
*TNF* rs361525	GG	37	7.9%	11	0%	p = 0.475	30	7.1%	18	5%	p = 1.008
	GA	7		0			5		2		
	AA	0		0			0		0		
*TNF* rs1800629	CC	42	2.3%	10	4.5%	p = 1.128	33	2.9%	19	2.5%	p = 1.146
	CT	2		1			2		1		
	TT	0		0			0		0		
*CXCL8* rs4073	TT	15	42%	1	50%	p = 0.501	11	45.7%	5	40%	p = 0.561
	TA	21		9			16		14		
	AA	8		1			8		1		
*CXCL8* rs2227306	CC	15	42%	1	50%	p = 0.501	11	45.7%	5	40%	p = 0.561
	CT	21		9			16		14		
	TT	8		1			8		1		
*CXCL8* rs2227307	TT	15	42%	1	50%	p = 0.501	11	45.7%	5	40%	p = 0.561
	TG	21		9			16		14		
	GG	8		1			8		1		
*CXCL8* rs188378669	GG	44	0%	11	0%	p = 1.000	35	0%	20	0%	p = 1.000
	GT	0		0			0		0		
	TT	0		0			0		0		
*IL1B* rs1143634	GG	27	20.5%	7	18.2%	p = 1.000	22	20%	12	20%	p = 1.000
	GA	16		4			12		8		
	AA	1		0			1		0		
*IL1B* rs16944	TT	5	71.6%	0	77.3%	p = 0.915	5	64.3%	0	87.5%	**p = 0.008**
	TC	15		5			15		5		
	CC	24		6			15		15		
*IL1B* rs1143639	GG	28	19.3%	6	22.7%	p = 0.768	21	21.4%	13	17.5%	p = 0.620
	GA	15		5			13		7		
	AA	1		0			1		0		
*IL1B* rs1143627	GG	5	70.4%	0	68.2%	p = 0.835	4	62.8%	1	82.5%	**p = 0.030**
	GA	16		7			18		5		
	AA	23		4			13		14		
*IL6* rs1800795	CC	12	46.6%	4	50%	p = 0.774	9	48.6%	7	45%	p = 0.718
	CG	23		3			18		8		
	GG	9		4			8		5		
*IL10* rs1800896	AA	13	41%	3	50%	p = 0.440	7	47.1%	9	35%	p = 0.215
	AG	26		5			23		8		
	GG	5		3			5		3		
*CFTR* F508del	del/del	11	53.4%	2	36.4%	p = 0.504	11	55.7%	2	45%	p = 0.279
	del/-	25		6			17		14		
	-/-	8		3			7		4		

Considering the fact, that analyzed changes formed in our study two haploblocks (in *CXCL8* and *IL1B* genes), an analysis of the haplotypes in the context of the course and manifestation of disease was performed. We proved, that only haplotype AC created by changes c.-118G > A and c.-598T > C in *IL1B* gene is significantly more often observed in group with severe course of CF in comparison with mild course (80% and 61.4%, respectively; χ ^2^ = 4.055; p = 0.03).

## Discussion

Because CF is a multifactorial, life-shortening disorder, the determination of SNPs that would affect the general phenotype or course of the disease is essential, but also challenging due to previous inconclusive results. So far, most of the CF studies were focused on searching modifier genes responsible for the severe pulmonary phenotype of the disease. In our investigation we have analyzed the impact of selected 12 potential candidates of modulator changes, rs4073, rs2227306, rs2227307 and rs188378669 in *CXCL8* gene *,* rs361525 and rs1800629 in *TNF* gene, rs16944, rs1143634, rs1142639 and rs1143627 in *IL1B* gene, rs1800795 in *IL6* gene and rs1800896 in *IL10* gene on CF phenotype in Polish patients, taking into account the severity of symptoms on the side of the digestive, but also, respiratory system. Our hypothesis was that candidate modulator changes may predict digestive character of CF.

We observed, that in most of our group of patients (80%) the dominating symptoms occurred from the respiratory system and only in 20% of CF patients from the digestive system. Severe character of lung disease, diagnosed based on the FEV1% values, was noted in 20 patients (36%) and mild in 35 individuals (64%). In those subgroups of patients, we have performed a correlation analysis with DNA changes. Obtained variant allele frequencies of analyzed genetic variants, did not much differ from reference values for European population in 1000 Genomes database, except change *TNF* c.-308C > T (rs1800629) which occurred in our patients group less often (2,7%) than in the database (13%). Also interesting is, that variant *CXCL8* c.91G > T, p.Glu31Ter (rs188378669) globally noted with variant allele frequency < 0.1%, was detected in our CF patients at level 0.9% (one heterozygote detected in a cohort of 55 individuals). In our previous study, we proved, that this variant is significantly more common in patients with inflammatory bowel disease (MAF = 2.12%, 15 heterozygotes detected in a cohort of 353 patients) compared to healthy Polish population (MAF = 0.25%, 1 heterozygote identified in a cohort of 200 individuals of Polish population), what may suggest its association with inflammatory diseases (unpublished data). Therefore, studies on a larger group of patients are undoubtedly necessary to verify the participation of this variant in CF, especially since there are no data on the relationship with this disease.

Our study revealed, that among all analyzed genetic changes two of them, c.-598T > C (rs16944) and c.-118G > A (rs1143627) located in the *IL1B* gene, are significantly associated with the severe character of lung disease in polish CF subjects. Allele C in *locus* -598 was observed with frequency of 87.5% in patients with severe lung disease compared to patients with mild lung dysfunction (64.3%, p = 0.008, OR = 3.88, C.I. = [1.351-11.190]), while allele A in *locus* -118 was observed with frequency 82.5% and 62.8% in both groups, respectively (p = 0.03, OR = 2.78, C.I. = [1.079-7.194]). We confirmed high LD between both changes (rs1143627 and rs16944) creating haplotypes AC, GT, GC and AT, where AC was significantly more often observed in subjects with severe course of CF in comparison to mild.

Our findings concerning the impact of polymorphism rs16944 on CF phenotype are consistent with those obtained by de Vries *et al.* ^18^ They also proved a significant correlation of the variant allele c.-598C of *IL1B* gene with severe pulmonary dysfunction in total of 152 Australian CF patients. Similarly, Levy *et al.* ^19^ have reported that *IL1B* constitutes a clinically relevant modulator of CF lung disease in the study conducted among American patients. However, in their research other SNPs, rs1143634 and rs1143639 demonstrated a consistent association with severe pulmonary phenotype.

In contrast to those results, Corvol *et al.* ^8^ did not find any correlation between variants c.-598T > C and c.-118G > A in *IL1B* gene and lung function assessed by spirometry in 329 Caucasian CF children from France and Germany. Additionally, they did not confirm any linkage disequilibrium between those polymorphisms.

Studies mainly highlight the relationship between lung disease in CF and *CXCL8* gene polymorphism. ^22^^–^^24^ IL8 plays a crucial role in the pathophysiology of inflammation of the airways in CF patients caused by a deficiency or absence of the CFTR protein. ^25^ Our study did not confirm this association among Polish patients.

We are aware of several limitations of our research. Our study cohort included only 55 patients and 50 controls. In the next step, verification of our results should be performed on a larger group of patients. This may be crucial in the case of rare variants, as c.91G > T, p.Glu31Ter (rs188378669) in *CXCL8* gene, candidate as a modifier of CF. Furthermore, other factors such as BMI, gender, or age of diagnosis was not taken into account in our statistical analyzes.

We should also highlight the strengths of our study. First, the study cohort was represented by detailed characterized patients and homogenous controls group. What is important, the effect of *CFTR* mutation F508del on the manifestation and course of CF in the studied patients was excluded because the frequency of mutations in the subgroups was similar.

Although this study does not indicate any modulators of digestive manifestation of CF, it constitutes the first report of genes predicting the course of this disease in the Polish population.

Recent studies indicate the important role of the microbiome in the course and manifestation of cystic fibrosis. Scientists underline that both, genotype and microbiome profiles are crucial interconnected factors in disease progression. ^26^

## Conclusions

Our data have shown, that from all analyzed pro-inflammatory cytokine genes, only *IL1B*, but not *TNF, CXCL8, IL6,* or *IL10* clearly play a crucial role in CF manifestation, determining the severe character of lung disease. This is a confirmation of major global results, as well as the first report concerning modulator genes of CF manifestation among Polish patients. Unfortunately, none of the analyzed genetic variants was found as predictors of digestive manifestation of CF disease, which may suggest the participation of also other modulator genes in the final phenotype of the disease.

## Data availability statement

All data underlying the results are available as part of the article and no additional source data are required.
