# Supplementary material for: IL1B gene variants, but not TNF, CXCL8, IL6 and IL10, modify the course of cystic fibrosis in Polish patients
Source: F1000Res. 2022 Nov 22;11:379. Originally published 2022 Mar 31. [Version 3] doi: 10.12688/f1000research.110472.3 (PMC11344199; doi:10.12688/f1000research.110472.3)
Supplement: Peer review history

# Reviewer report: Reviewer response for version 3

**DOI:** 10.5256/f1000research.141310.r279792

**Published:** 2024-08-23

**Authors:** Agnieszka Swiatecka-Urban

**Affiliations:** 1 University of Virginia, Charlottesville, Virginia, USA

The authors examined the association of polymorphic variants in genes from the inflammatory pathway with pulmonary or digestive manifestation of cystic fibrosis (CF), and the severity of CF lung disease.

 It is an interesting and important study. However there are major issues that should be addressed before the work is accepted.

 Major:

 The size of the cohort. The authors should provide the rationale for the small number of patients enrolled in the study. 

 Variable genetic background: Only 23.6% of patients were homozygous for F508del. The remaining patients were compound heterozygotes or had undisclosed CFTR genotypes. The non-homogenous genetic background in the CFTR gene invalidates the conclusion that the gene modifiers were responsible for the severity of pulmonary and digestive CF disease. If the sample size was larger, a multivariate analysis could be used to tease out potential associations.

 Healthy controls: The purpose of including healthy controls needs to be clarified and discussed in the manuscript. 

 The authors emphasize the strengths of the study but neglect to mention the study limitations.

 Minor:

 There are many typographical and syntax errors. I suggest that the manuscript is reviewed by a professional English language interpreter.

Is the work clearly and accurately presented and does it cite the current literature?

Partly

If applicable, is the statistical analysis and its interpretation appropriate?

Partly

Are all the source data underlying the results available to ensure full reproducibility?

Yes

Is the study design appropriate and is the work technically sound?

Partly

Are the conclusions drawn adequately supported by the results?

Partly

Are sufficient details of methods and analysis provided to allow replication by others?

Partly

Reviewer Expertise:

NA

I confirm that I have read this submission and believe that I have an appropriate level of expertise to confirm that it is of an acceptable scientific standard, however I have significant reservations, as outlined above.

# Reviewer report: Reviewer response for version 2

**DOI:** 10.5256/f1000research.134254.r151823

**Published:** 2022-10-24

**Authors:** Daniel J. Smith

**Affiliations:** 1 Faculty of Medicine, The University of Queensland, Brisbane, Qld, Australia

The article aims to determine whether there are association with polymorphisms in the genes for common pro-inflammatory cytokines and severity of CF lung and GI manifestations.

 The major findings, in general, are supported by findings from studies in other populations.

 Major comments:

- The population size is relatively small for a study of this type and runs the risk of type 2 error (falsely exclude the null hypothesis).
- The definition of severe lung disease is based purely on lung function. However, this does not take into account age, i.e. a patient who has an FEV1 of 50% at the age of 20 years of age has a more severe phenotype compared to a patient who has an FEV1 of 50% of predicted at the age of 50 years (it is likely the 50 year old had a substantially higher FEV1 %predicted when they were 20 years old) ^1^. There are other tools available in the literature for classifying the severity of lung disease taking age into consideration.
- Lung function and nutrition in people with CF are not independent. Often patients with the most severe lung disease, also have the worse nutritional status. Greater detail needs to be provided on how patients were classified to a pulmonary or GI predominant phenotype. This classification is subject to bias. It should be stated whether the specialists determining the patients phenotype were also involved in analysing the results of genotype assessment and whether all phenotyping was completed before the genotype results were available.
- Information should be provided on the rates of the different polymorphism among people with different CF genotypes. If certain polymorphisms are over represented in people homozygous for the F508 mutation. It may be the CF genotype and not the polymorphism that explains the difference in phenotype.

Minor comments:

- Lung function will vary with clinical status. Were all patients clinically stable at time of recruitment? If not, best lung function in the last 12 months may be a better marker of severity of lung function impairment.

Is the work clearly and accurately presented and does it cite the current literature?

Yes

If applicable, is the statistical analysis and its interpretation appropriate?

Yes

Are all the source data underlying the results available to ensure full reproducibility?

Yes

Is the study design appropriate and is the work technically sound?

Yes

Are the conclusions drawn adequately supported by the results?

Yes

Are sufficient details of methods and analysis provided to allow replication by others?

Yes

Reviewer Expertise:

Cystic Fibrosis Physician. Published in the field of CF modifier genes during PhD studies.

I confirm that I have read this submission and believe that I have an appropriate level of expertise to confirm that it is of an acceptable scientific standard, however I have significant reservations, as outlined above.

## References

[1] : Classifying Severity of Cystic Fibrosis Lung Disease Using Longitudinal Pulmonary Function Data. *American Journal of Respiratory and Critical Care Medicine* .2006;174(7) : 10.1164/rccm.200512-1919OC 780-786 10.1164/rccm.200512-1919OC PMC264806216858011

# Response

**Published:** 2022-11-18

**Authors:** Oliwia Zakerska-Banaszak

**Affiliations:** Institute of Human Genetics PAS, Poland

Dear Dr. Daniel J. Smith,

 Thank You very much for reviewing our manuscript and for all valuable comments and suggestions, which we have analyzed step by step.

 We response accordingly to Your comments:

- The population size is relatively small for a study of this type and runs the risk of type 2 error (falsely exclude the null hypothesis).

We agree, the study group is not big, it consists of only 55 patients and of a proportionally big control group of 50 healthy Polish subjects, and we are aware of this limitation of our research. We believe, we have collected as many patients, as we can in this study period and during this collaboration with the Department of Pulmonology, Allergology, and Lung Oncology, Poznan University of Medical Sciences. On the other hand, this group is well-characterized and enrolled in accordance with the assumptions of the study.

- The definition of severe lung disease is based purely on lung function. However, this does not take into account age, i.e. a patient who has an FEV1 of 50% at the age of 20 years of age has a more severe phenotype compared to a patient who has an FEV1 of 50% of predicted at the age of 50 years (it is likely the 50 year old had a substantially higher FEV1 %predicted when they were 20 years old) ^1^ . There are other tools available in the literature for classifying the severity of lung disease taking age into consideration.

Thank You very much for this comment. We agree, in the literature, there are available different tools for classifying the severity of lung disease. In our study, we were based also on the professional literature (Yankaskas JR et al. Cystic fibrosis adult care: consensus conference report. *Chest* 2004;125:1-39), where the classification was determined from FEV1 only, as follows: 40-69% mild, and <40% severe. Nevertheless, due to the importance of age in this classification and accordingly to your suggestion, we took age into consideration in both groups and compared them in this regard. For this purpose statistical analysis (unpaired t-test) was performed. Results revealed that the “severe” group of patients did not differ significantly in age from patients in the “mild” group (mean age was 27.11 and 30.75, respectively; *p*-value = 0.055).

 Therefore, the reference (numbered 21) and the information in the text was added (page 6) :

*“The “severe” group of patients did not differ significantly in age from patients in the “mild” group (mean age was 27.11 and 30.75, respectively; p-value = 0.055)*” *.*

 In the future study, we will be careful and meticulous in classifying patients with CF to take age into account.

- Lung function and nutrition in people with CF are not independent. Often patients with the most severe lung disease, also have the worse nutritional status. Greater detail needs to be provided on how patients were classified to a pulmonary or GI predominant phenotype. This classification is subject to bias. It should be stated whether the specialists determining the patients phenotype were also involved in analysing the results of genotype assessment and whether all phenotyping was completed before the genotype results were available.

Thank You for this important remark. Of course, nutritional status is correlated with the severity of the disease. We can observe (based on BMI) this association in our study – in the “mild” group 26% of patients were malnourished, while in the “severe” group, 60% of patients.

 Patients were classified by the specialists from the pulmonology field conducting the patients, based on the long-term observation of the patient and personal clinical interview with patient, to a pulmonary or GI predominant phenotype (we did not designate groups of pulmonary or digestive type of the disease, we only assessed that the dominant symptoms in a given patient were from the respiratory or digestive system, which does not mean that it was not from the other system). To the GI predominant phenotype were enrolled patients with the coexistence of at least two of listed conditions: 1) diabetes or glucose metabolism impairment, 2) pancreatic insufficiency, 3) liver disease or cirrhosis, 4) nagging pain or dysfunction of the digestive system. All patients with GI predominant phenotype represented “mild” lung impairment. Specialists determining the patients phenotype were not involved in the analysis of genotype assessment, of course. Results of genotyping did not influence the assessment of phenotypic description.

 Therefore, in the text on page 6, the additional information was added to clarify:

 “ *To the digestive predominant phenotype were enrolled patients with the coexistence of at least two of listed conditions: 1) diabetes or glucose metabolism impairment, 2) pancreatic insufficiency, 3) liver disease or cirrhosis, 4) nagging pain or dysfunction of the digestive system. All patients with GI predominant phenotype represented “mild” lung impairment. Specialists determining the patients phenotype were not involved in the analysis of genotype assessment. Results of genotyping did not influence the assessment of phenotypic description”*.

- Information should be provided on the rates of the different polymorphism among people with different CF genotypes. If certain polymorphisms are over represented in people homozygous for the F508 mutation. It may be the CF genotype and not the polymorphism that explains the difference in phenotype.

Thank You for this valuable comment. Accordingly to Your suggestion, we provide information about CF genotypes among patients group in the Table 4 containing analysed polymorphisms.

- Lung function will vary with clinical status. Were all patients clinically stable at time of recruitment? If not, best lung function in the last 12 months may be a better marker of severity of lung function impairment.

Thank you very much for this valuable remark. Of course, all the patients were clinically stable at the time of study recruitment.

 Hence, additional information was added in the text on page 6:

*“Patients were divided in the context of lung function impairment, based on the FEV1% values, **while they were clinically stable**, 1 - within the norm (FEV1% ≥ 70) and mild pulmonary obstruction (FEV1% 40-70) (35 subjects in total), 2 - severe pulmonary obstruction (FEV1% ≤ 40) (20 subjects in total)”.*

 We hope, our responses improve the understanding of the manuscript.

 Yours sincelery,

 Oliwia Zakerska-Banaszak

# Reviewer report: Reviewer response for version 1

**DOI:** 10.5256/f1000research.122087.r135127

**Published:** 2022-05-16

**Authors:** Tadeusz Przybyłowski

**Affiliations:** 1 Department of Internal Medicine, Pulmonary Diseases and Allergy, Medical University of Warsaw, Warsaw, Poland

This is a very interesting paper in which the authors decided to evaluate whether selected polymorphic variants in genes encoding selected inflammatory mediators can predict pulmonary or digestive manifestation of cystic fibrosis and severity of lung involvement. Using advanced genetic engineering techniques they have genotyped 12 variants in TNF, CXCL8, IL1B, IL6 and IL10 genes in a group of 55 Polish patients with cystic fibrosis and 50 healthy controls. The most important outcome was the finding of significant differences in the frequency of two promoter variants of IL1B, rs1143627 (c.-118G > A) and rs16944 (c.-598T > C) in relation to the severity of lung involvement. It was also observed that AC haplotype was significantly more frequent in the group of patients with more advanced lung damage than in the group with milder disease progression. These results allowed the authors to conclude that genetic variants, rs1143627 and rs16944, of IL1B may be used as predictors of more severe respiratory involvement in the course of cystic fibrosis. Additionally, it should be noted that this is the first study of its type performed in a group of Polish patients.

 The work was prepared very carefully, but in my opinion, 2 issues need re-evaluation.

- The way of classifying patients into different stages of disease severity on the basis of FEV _1_ is not very clear to me. Why the cut-off points of 70% and 40% of predicted were used?. In this part of method description there should be information if it was arbitrary classification or if it is consistent with the generally accepted system of grading, and in this case reference to an appropriate publication should be given.
- The paragraph concerning the occurrence of PA and MRSA in the studied group does not fit into the subject of the paper, does not add any new information, and may be omitted in the final version.

Is the work clearly and accurately presented and does it cite the current literature?

Yes

If applicable, is the statistical analysis and its interpretation appropriate?

Yes

Are all the source data underlying the results available to ensure full reproducibility?

Yes

Is the study design appropriate and is the work technically sound?

Yes

Are the conclusions drawn adequately supported by the results?

Yes

Are sufficient details of methods and analysis provided to allow replication by others?

Yes

Reviewer Expertise:

Lung diseases

I confirm that I have read this submission and believe that I have an appropriate level of expertise to confirm that it is of an acceptable scientific standard.

# Response

**Published:** 2022-05-19

**Authors:** Oliwia Zakerska-Banaszak

**Affiliations:** Institute of Human Genetics PAS, Poland

Dear Reviewer, Prof. Przybyłowski,

 We thank you very much for your comments and all the valuable suggestions concerning our manuscript. 

 According to Your questions:

1. The way of classifying patients into different stages of disease severity on the basis of FEV _1_ is not very clear to me. Why were the cut-off points of 70% and 40% of predicted were used?. In this part of the method description there should be information if it was arbitrary classification or if it is consistent with the generally accepted system of grading, and in this case reference to an appropriate publication should be given.
2. The paragraph concerning the occurrence of PA and MRSA in the studied group does not fit into the subject of the paper, does not add any new information, and may be omitted in the final version.

Our response:

1. **In the literature on cystic fibrosis, disease severity is determined from FEV1 as follows: FEV1> 70% severe, 40-69% moderate, and <40% severe. Such a division was presented, inter alia, in the work: Yankaskas JR. Cystic fibrosis adult care: consensus conference reprt. chest 2004; 125: 1-39.**
2. ** The paragraph concerning the occurrence of PA and MRSA  was omitted.**

Thank You very much. We hope our revised version of the manuscript will be correct now.

 Your sincerely,

 Oliwia Zakerska-Banaszak
